# The Children's Ward in Design and Decoration

**Published:** 1912-06-29

**Authors:** 


### THE MAKING OF A MODERN HOSPITAL.*

### The Children's Ward in Design and Decoration.

Properly speaking, the children's ward belongs to the group of special departments. It should be regarded as a special unit and treated as such. In most hospitals, however, where, for various reasons, it has been found impracticable to separate it so sharply from the other wards, the children's ward is merely a slightly more or less elaborated ordinary ward which is given over to the child patients. In many cases, unfortunately, it is argued that because the patients are small, children in fact, they need less attention, less consideration from the ward constructor's point of view than do adult patients. The results of this amazing presupposition are to be seen in the children's wards of several of our London and provincial hospitals. These, which may be called examples of the old-fashioned style of children's ward, are usually merely on the same lines as the wards for grown-ups. The gross changes observable are that they are furnished with cots instead of with beds, and that they are usually floridly coloured with wall paintings that are supposed to be decorative. One might easily fill a page with the enumeration of the faults of these juvenile wards, but the whole indictment against them is compressed into a line when it is stated that they are ordinary wards which have been "adapted" for the use of child patients on lines which are no longer regarded as scientific and defensible.

### The Need for Collaboration.

We have urged in previous articles the importance of bearing in mind the ordinary principles of physiology and biology, no less than the essentials of hygiene and ventilation, engineering, and drainage, in the construction of a modern hospital. Manifestly an architect, however ambitious he may be, and however catholic the scope of his reading and requirements, cannot be expected to master all these subsidiary details. Therefore it is evident that he must call in, if he wishes to be a good hospital architect, the experience and expert medical knowledge of the hospital doctor. And it is well to lay stress on a point very often overlooked—namely, that the specialists in various departments which make up the hospital should be consulted in regard to their departments as well as with reference to the whole plan of construction. We do not venture to suggest that their suggestions should be immediately and wholly followed. Very often specialists have cramped ideas; they can only see their own side of the question at issue and fail to correlate facts which the architect and the medical coadjutor in the construction must constantly bear in mind. But here and there in hospitals in which the special departments are, from many points of view, admirable in detail, we find that they provide many objectionable points when the whole plan is taken into consideration, since allowance has not been made for correct approaches and co-operation with other departments. In one modern hospital, which is otherwise a beautiful model, the psychiatric department is placed in close proximity to the pathological department with its mortuary—an arrangement which can only be styled most undesirable in any circumstances. We lay stress on this matter of co-operation and co-suggestion because its importance is accentuated in the construction of the children's ward. One or two points, for example, should always be remembered by anyone who visits such a ward and who wishes to judge of its goodness or badness on technical lines. These points are often forgotten, even by authorities of established repute who still adhere to the old-fashioned rules with regard to ventilation and lighting of children's wards. Probably what we shall have to say in this article may sound strange to many who hold to these views, but we can only plead that our remarks are in consonance with the best modern teaching, and that, if here and there we show a tendency to go further than most authorities would care to go, the advance is in the right direction.

### Forgotten Facts About the Child.

It is a common fallacy to suppose that a child needs less air, less oxygen, less light than an adult. Weight for weight the average healthy child needs at least one-half more of these indispensable requirements than does the average healthy adult. Formerly it was supposed that young plants required less air space and nourishment than older plants; now we know that they need relatively more. The reason is that in the young organism metabolism proceeds with comparatively greater rapidity than in the adult, and with far greater speed than in the aged. Children, for instance, need a richer dietary than adults—again basing the comparison on body weight. They also need more tissue food in the shape of oxygen. To give to a children's ward, then, an air area which is calculated on the assumption that the child patients need far less air than do the adult patients is to err on the side of parsimony. The cubic content demanded by the child should not be less than that given to the adult. The regulations for ventilation and the renewal of the air supply must be perfect. Every attempt must be made to flush the ward half-hourly at least so that the air in every cranny and nook is renewed. The child patient must have pure air and the requisite combination of oxygen and nitrogen, neither too much of the one nor too little of the other. For he is doubly sensitive to the subtle detrimental action of exhalations. We do not yet know why exhalations exert this effect, but we know that the air of an overcrowded ward is poisonous, so much so that a condensation of the water vapour which it contains if injected into the animal's veins will kill a rabbit. What matters is not total volume of air but the free supply of fresh air, since it is air in motion and not stagnant air that is required. It is easy, therefore, to understand the absolute necessity for purity and freshness of the

* Previous articles appeared on Oct. 7, 14, 21, 28, Nov. 4, 18, Dec. 9, 30, Feb. 10, March 16, April 6, 20, May 4, 18, June 1, 15.

air. And it is equally easy to realise the importance of equability. The ward must be kept at a uniform temperature, and for that purpose natural ventilation with open fires—always properly screened—will probably be found the most successful means of heating and ventilating. The open fire is also much more attractive than the radiator. There is no real objection to good radiators, except that they are apt to get clogged and generally give rise to a purely subjective feeling of chilliness. This is not because they are less reliable than the open fire, but simply because they lack the suggestive effects of the fire—a suggestive effect which children appreciate far more than do adults. The lighting of the ward must be even, free, and sufficient. Here more than anywhere else in the hospital it is necessary that every inch of floor space shall for a portion of the day be washed in direct sunlight if the sun is available.

### Walls and Wall-papers, Paints and Flat Finishers.

The walls and ceiling of the ward must be attended to on the general lines already laid down in previous articles. Impermeability is an absolute necessity. The use of wallpapers is quite indefensible, even when they are of the so-called washable type, the reason being that the paste and not the paper is unhealthy and a breeding-ground for pyogenic bacteria. Cold-water paints are inadvisable, as they soon scale and cannot be depended on, while the glue that most of them contains is objectionable since it generates a musty odour in course of time. Mr. Gardner, an American authority, referring to the use of such paints in hospital wards, remarks: "It is well known that meats, beverages, and other sensitive foodstuffs placed in rooms decorated with cold-water paints are liable to absorb odours which ruin their taste. There have recently appeared on the market paste-paints which contain a rosin varnish binder mixed with glue, dextrin, and other water soluble materials of an adhesive nature. . . . Paints of this type are even more dangerous to use than ordinary calcimine." The same authority lays down the following good rules for testing the merits of a wall paint suitable for hospital use: "The paint . . . must be of such a nature as to form on drying a hard, elastic, and impervious surface that may be washed with water or antiseptic solutions or subjected to the fumes of formaldehyde or sulphur dioxide. The pigments in such paints must be of a permanent type, unfading, non-darkening, and unaffected, even when tinted to the most delicate hue, by the caustic lime that may exist in cement and plastered walls." He recommends the use of flat-finishers for the ward walls. If these be used it is as well to avoid the varieties which contain decomposable oxides. The best kinds are those which are composed chiefly of the stable oxides of barium, zinc, and silicon. The painting of the floor, where a composition floor is used, must also be carefully considered.*

### The Child's View on Wall Decorations.

With regard to the decoration of the walls, pictures and framed engravings are usually undesirable for the same reason that all appendages and fittings which require to be taken down for cleaning purposes are undesirable. If pictures be used they must be painted on the wall. It is questionable if the "modern art wall" decorations seen in some children's wards is in the interests of the little patients. The colours are generally loud and glaring and the scheme of decoration is usually much more suitable for a day room or nursery than for a sickroom. The artist who painted such a dado as exists in one London hospital, on which the weird adventures of Jack the Giant Killer are represented in hideously realistic fashion, and the good ladies who paid for the painting, would probably object most strongly to being kept in such a room when they were feverish. Everyone who has had experience, actual nursing experience, in a children's hospital is aware how sensitive the little patients are to outside influence, and especially to ocular impressions which adults would, almost without exception, disregard. A delirious child or a nervous patient, new to the ward, finds a haunting terror in the figure decorations on the walls. That this is no exaggeration has been proved over and over again. Figure decorations are unsuitable in a sickroom no matter how artistically they may be designed. They suggest action, and in many cases the pictures of human beings and animals on the wall have a very curious psychological effect upon young patients which must be regarded as distinctly detrimental.

Must we then dispense entirely with wall decoration in the children's ward? By no means. The object of such decoration is to soothe, compose, and please, and so long as the decorator bears in mind the fact that the essential thing above all is harmony of colour and form, no great exception, beyond the argument that colour must necessarily stimulate a sick child under certain conditions, can be taken. Floral mural decorations are far preferable to figure paintings; geometric designs and strong contrasts should be avoided. In any case where a floral or decorative dado is used the cots should be so placed that the children do not face the painting when they lie down. A more attractive decorative effect is usually secured by coloured-glass slips in the lower windows; the reflections are always pleasing to the children, and somehow do not appear to have the stimulative effects which are produced by fixed colours.

* In a former article ("The Ward Ceiling, the Roof and Walls," The Hospital, June 15, pp. 273-274) we have gone fully into the question of paints and washable distempers. Since then we have been able to study the effects of a few new varieties which appear to us to merit a trial in English hospitals. One of these is the "Abwaschbare Eck. W. Farbe" (Ecks W. Washable Wall Paint) supplied by the firm of M. Eck, of Oberursels (Taunus). This is a compromise between oil and lime-wash paint, and is suitable for plaster, wood, or metal work. It has the advantage of being economic, lasting, and impermeable. Other useful preparations are Meissner's Lead-free Paint (*Bleifreie Anstrichmassen*), suitable for iron and zinc work; the lacquer paint *chrotogen* of Dr. Münch; and the smoothly drying Selhamin-paint manufactured by Hecht, Katzenberger and Co., of Cologne. Equally excellent paints are supplied by the Kitzinger Colour Manufactory at Kitzingen-am-Main.

